# Effect of Multimodal Intervention (computer based cognitive training, diet and exercise) in comparison to health awareness among older adults with Subjective Cognitive Impairment (MISCI-Trial)—A Pilot Randomized Control Trial

**DOI:** 10.1371/journal.pone.0276986

**Published:** 2022-11-03

**Authors:** Prasun Chatterjee, Deepa Anil Kumar, Sana Naqushbandi, Preeti Chaudhary, Preetika Khenduja, Swati Madan, Sobia Fatma, Maroof A. Khan, Vishwajeet Singh

**Affiliations:** 1 Department of Geriatric Medicine, All India Institute of Medical Sciences, New Delhi, India; 2 Department of Biostatistics, All India Institute of Medical Sciences, New Delhi, India; Prince Sattam Bin Abdulaziz University, College of Applied Medical Sciences, SAUDI ARABIA

## Abstract

**Introduction:**

This study was aimed to investigate the effect of multimodal intervention on the cognitive functions of older adults with subjective cognitive impairment (SCI).

**Materials and methods:**

Sixty subjects were randomized 1:1:1:1 to receive either computer based cognitive therapy (CBCT) or CBCT+Mediterranean equivalent diet (MED) or CBCT+MED+ Exercise regime and the control group. The intervention group received supervised CBCT twice a week to have 40 sessions, each of 40 minutes duration, and/ or supervised aerobic and resistive exercise twice a week for 24 weeks and or MED at home under the supervision of a dietician. The control group was provided with health awareness instructions for brain stimulating activities such as sudoku, mental maths, and learning music and new skills.

**Results:**

Cognitive functions which was the primary outcome measure were assessed using the Post Graduate Institute Memory Scale (PGI-MS), and Stroop Colour and Word Test at baseline and after 6 months intervention period. As assessed by the PGI-MS, there was significant improvement in domains such as mental balance, attention and concentration, delayed recall, immediate recall, verbal retention of dissimilar pairs, Visual retention, and total score both in the unimodal and multimodal intervention groups. However, the improvement was observed to be the highest in the multimodal intervention group as compared to unimodal group. All the participants completed the trial.

**Conclusion:**

This pilot randomized control trial indicated that multimodal intervention could be an effective non-pharmacological intervention in individuals with SCI for improving their cognitive functions.

## Introduction

Subjective Cognitive Impairment (SCI) is defined as a self-experienced persistent decline in cognitive ability in comparison with the previously normal status of the subject without objective cognitive decline [1]. However, recent epidemiological evidence suggests that SCI subjects have difficulties in complex attention, episodic memory, recent memory, executive functioning and are at 4.5 times increased risk of developing dementia in three to seven years, as compared to their cognitively normal counterparts [2]. An early intervention at SCI stage could be a definite opportunity to prevent the progression of cognitive impairment and related complications.

Non-pharmacological interventions (NPI) have been reported to be a viable alternative for persons with SCI [3] even though there is no evidence-based consensus treatment for SCI [4]. Previous NPI interventions on older adults with cognitive impairment are either unimodal (psychotherapy) or applied in advance stage of the continuum of dementia [5].

As per evidence, computer based cognitive training has shown promising results in cognitive stimulation through various mechanisms [6]. RehaCom, a German software, works for the rehabilitation of cognitive disorders, memory, attention, concentration, executive functioning and visuo-motor abilities, might be one of the best fitted Computer based cognitive training (CBCT) software for SCI in older adults [7]. This software has about 30 modules available in 27 other native languages. RehaCom has a wide range of applications and can be applied on people with acquired brain injury, like stroke, in children with Attention deficit hyperactivity disorder, depression etc [8, 9].

It has been reported that regular exercise, both aerobic and resistive, have protective value against oxidative damage, neuro-inflammation, and amyloid deposition, and it also enhances neurogenesis, synaptic plasticity, cerebral perfusion and mitochondrial function of the brain cell [10]. Similarly, the Mediterranean diet has been reported to have an enhanced effect on cognitive abilities [11]. Traditional Indian diet is different from the Mediterranean diet. However, incorporating the socio-culturally modified Mediterranean equivalent diet (MED) could be tried in older Indians with SCI.

Keeping in view of the above-mentioned facts, we aim to examine the effect of CBCT using RehaCom software, MED and exercise in comparison to health awareness (Control) in prevention of cognitive decline in older adults with SCI. It was hypothesized that a combination of CBCT and healthy lifestyle (MED and exercise) would help in preventing the progression of cognitive decline in SCI Subjects.

## Materials and methods

Study participants: The study was a randomized controlled trial conducted from November 2018 to November 2020. A total of 100 individuals who attended the out-patient department of the Department of Geriatric Medicine were contacted, of which 80 fulfilled the inclusion criteria. Out of 80, 20 individuals refused to take part in the study. Consequently, 60 consenting older adults were included for randomization after obtaining informed written consent. The study was conducted according to the declaration of Helsinki, and the protocol was approved by the Institute Ethics Committee of the All India Institute of Medical Sciences (AIIMS), New Delhi (IEC-242/05052017). The trial was registered under Clinical Trials Registry-India (CTRI/2018/10/016237).

Inclusion criteria: Consenting older adults aged 60 years and above having minimum education of 10^th^ grade with a Clinical Dementia Rating (CDR) score = 0 was included in the study. The exclusion criteria were CDR ≥ 0.5, Geriatric depression Scale (GDS) (>4) and severe musculo-skeletal and neurological impairment [12].

### Randomization

**Fig 1:**
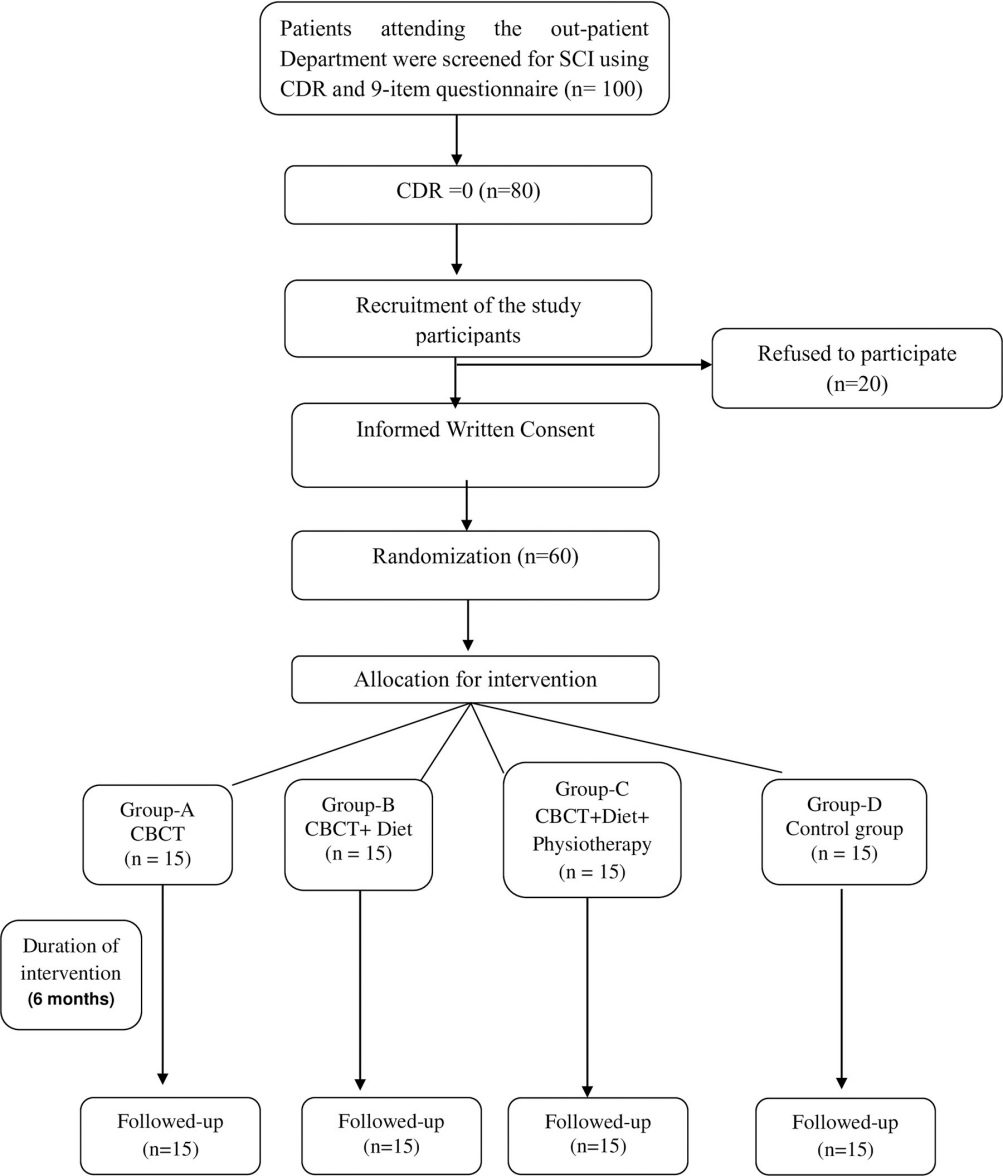
Study design.

A computer based random list was generated for allocation to each intervention. Allocation of the participants to the intervention group was performed using sequentially numbered, opaque, sealed envelopes. The participants were allocated into four groups (Group A, B, C and D) as follows; Group A: Cognitive Training; Group B: Cognitive Training + Diet; Group C: Cognitive Training + Diet + Exercise (CDE) and Group D: Health Awareness, having 15 participants in each group (Fig 1).

### Intervention package

#### CBCT using RehaCom

RehaCom software was used to train different areas of cognitive functions starting at a low level of difficulties; the participants were confronted with more complex tasks at higher levels. There were 29 types of tasks under different cognitive domains covering attention, memory, visuo-spatial functioning and executive functioning. All the tasks were keyboard based and the clients were given instructions prior to the task. Cognitive training was carried out under the guidance of an experienced clinical psychologist.

#### Mediterranean Equivalent Diet (MED)

The Mediterranean diet plan from the Finnish Geriatric Intervention Study to Prevent Cognitive Impairment and Disability (FINGER) [13] trial was modified by experts primarily based on the Indian Council of Medical Research and National Institute of Nutrition Recommendations [14]. The diet regime comprised 20% of Daily Energy (DE) from proteins, 25% of DE from fat (<5% from saturated plus trans fatty acids, 0% from MUFA, and 20% from PUFA which includes 2.5–3g/day of omega-3 fatty acids), 55% of DE from carbohydrates (<10% from refined sugar), 30–35 g/day of dietary fibers, less than 5 g/day of salt, and no alcohol.

#### Exercise regime

A customized exercise module (aerobic and resistive) was developed with the help of experts from multiple departments at AIIMS, New Delhi. The in-person exercise regime was conducted under the guidance of an experienced physiotherapist. Aerobic exercises started with a warm-up for 10 min, comprising walking at comfortable speed followed by 20 min treadmill walk at 40% Heart Rate (HR) Max in the first month, progressing to 60% HR Max for another two months and 70%-80% HR Max for the last three months. Resistance training started with a warm-up for 10 min consisting of stretching exercises followed by exercises for upper and lower extremity and core strengthening. The training included two sets of 8–15 steps for each exercise with 5 min of rest interval in between the sets. Resistance training was given at 40%-50% Repetition Maximum (RM) for the first month, progressing to 60% RM for the next two months and 70% - 80% RM for the last three months. The participants practiced supervised exercise 2 times per week for 6 months.

Participants in the control group were provided with health awareness instructions for brain stimulating activities such as sudoku, mental maths, learning music and new skills. Their activities were monitored both telephonically and by bimonthly visit to the department.

Feasibility study was conducted on ten older adults with SCI who were not included in the main study. The feasibility study revealed that older Indians, especially those with less than 10 years of education, faced difficulty in performing various complex tasks as RehaCom was originally developed in Caucasian population. Ten participants who volunteered for the feasibility study also intervened using a modified diet regime and customized exercise protocol with 100% compliance and no side effects.

#### Main study

The participants took two sessions of computer based cognitive therapy (CBCT) per week under the supervision of Psychologist, to have 40 sessions within 24 weeks. Each session was of 40 minutes duration. level of difficulty was increased in each training task according to the performance of the study participants. Make up sessions were given to the participants who missed the sessions due to any reason.

Additionally, group B participants practiced MED at home, which was observed by the project dietitian by telephone every week and physically at the hospital once per month using diet recall chart. Group C received all three intervention modalities.

In addition, members of group B engaged in MED at home, which was observed by the project dietitian by telephone every week and physically at the hospital once per month using a food recall sheet. Similar to group B, group C also got all three intervention modalities.

#### Outcome measures

Cognitive functions which are the primary outcome measures were assessed using the following tools;

The Post Graduate Institute Memory Scale (PGI-MS), a validated tool in the Indian population [15] was applied to measure verbal and non-verbal memory. It consisted of 10 sub-tests, such as remote memory, recent memory, mental balance, attention and concentration, delayed recall, immediate recall, retention of similar, dissimilar pair, visual retention, and recognition.

Stroop Colour and Word Test [16] was used to measure cognitive flexibility and response inhibition by the relative speed of reading the names of colours. The T-score of word, colour and colour word less than 40 is deficit in cognitive processes. A low word score indicated poor dominance for reading skills and low color scores suggest colour-blindness.

While episodic memory of the study participants was assessed using the remote memory domain of the PGI Memory scale, visuo-spatial memory and working memory were assessed by the visual retention and immediate memory domains respectively.

The participants were assessed with similar assessment tools at baseline and after six months of the completion of the training.

#### Statistical methods

Quantitative variables obtained in the study were presented as mean (SD) and/or median (range) according to the distribution of the data and qualitative variables were presented as number (percentage). To find the association between categorical variables, Chi square test or Fisher’s exact test was used according to the frequency distribution of the data. One-way ANOVA with bonferroni correction/Kruskal-Wallis test followed with Dunn’s test was used to assess statistical differences in characteristics for quantitative variables. Paired t-tests were used to assess the pre and post intervention differences within the group for quantitative variables, with Mc Nemar’s test used for qualitative variables. Intention to treat approach was adopted for the analysis as no violation in protocol was observed. A *p*-value < 0.05 was considered as statistically significant. Statistical software STATA/SE (Ver 14.2) was used for the analysis.

## Results

### Baseline comparisons demographic profile and outcome variables

Out of 60 older adults, 63.33% were males and 36.67% were females. The distribution of male participants in group A, B, C and D were 73.33%, 53.33%, 66.67% and 60% respectively (*p* = 0.697). The mean age of the participants was 68 ± 6.46, 68.93 ±5.27, 65.2 ± 3.73 and 71.07±6.93 years (*p* = 0.055) in groups A, B, C, and D respectively. The analysis showed that there were no significant differences in the outcome variables between the groups at the baseline except in one of the domains of the PGI memory scale namely, recognition (Table 1).

**Table 1:** Baseline characteristics of the study participants.

Variables	Group A	Group B	Group C	Group D	*p-* value
	(n = 15)	(n = 15)	(n = 15)	(n = 15)	
Male	11	8	10	9	0.697
Gender					
Female	4	7	5	6	
Age ± SD (years)	68 ± 6.46	68.93 ± 5.27	65.2 ± 3.73	71.07±6.93	0.055
Remote Memory	6.73±0.79	6.73±0.59	6.8±0.77	6.73±0.59	0.991
Recent Memory	4.87±0.35	4.73±0.70	4.93±0.25	4.73±0.59	0.631
Mental Balance	7.6±1.59	7.86±1.18	7.6±1.35	7.2±1.32	0.616
Attention Concentration	6.93±2.15	9.73±4.36	8.93±2.96	9.06±4.60	0.193
Delayed Recall	8.4±2.09	7.73±1.79	8.73±1.5	8.2±2.27	0.564
Immediate Recall	10.26±1.27	8.6±2.12	9.8±2.14	10.13±1.12	0.058
Retention of Similar Pairs	4.6 ± 0.63	4.6±0.73	4.73±0.59	4.53±0.74	0.876
Retention of Dissimilar Pairs	11.67±3.98	11.07±2.49	11.33±3.94	12.27±3.13	0.797
Visual Retention	9.13± 3.4	10.6±1.72	10±3.16	9.67±2.61	0.543
Recognition	9.8±0.41	9.2±1.26	9.93±0.26	9.73±0.59	0.046
PGI-MS Total	80±7.91	80.93±6.52	82.8±7.34	82.27±5.6	0.677
T-score word by deviation score	23.67±7.44	29.53±11.11	21.06±8.61	27.53±11.64	0.094
T-score colour by deviation score	25.33±12.45	32.27±12	24.73±8.51	23.47±8.83	0.112
T-score colour- word by deviation score	37.6±7.62	38.46±7.26	33.67±6.60	36.73±6.35	0.267

#### Post intervention

It was noted that the total score of PGI-MS was significantly different between groups A and C (*p* = 0.005), A and D (*p* = 0.036), B and C (0.001) and C and D (*p < 0*.*001*). The improvement was also observed in domains such as mental balance (*p* = 0.002), attention and concentration (*p* = 0.008), immediate recall (*p* = 0.017), and visual retention (*p* = 0.028). (Table 2). Immediate recall and visual retention were found to be significantly different between groups B and C (*p* = 0.032) and C and D (*p* = 0.023) (Table 2). Further, among the study group, a significant improvement was observed in working memory (*p* = 0.017) and visuo-spatial memory (*p* = 0.028) (Table 2). The difference was found to be significant between group C and D (Table 2). The details on the inter-group comparisons have been presented in Table 2.

**Table 2:** Intervention effects across groups.

Variables	*p*-value						
	Overall *p*-value	A *Vs B*	A *Vs C*	A *Vs D*	B *Vs C*	B *Vs D*	C *Vs* D
Mental Balance	0.002	>0.999	>0.999	0.040	>0.999	0.009	0.004
Attention and Concentration	0.008	>0.999	0.008	>0.999	0.228	0.316	0.001
Immediate Recall	0.017	0.145	>0.999	0.664	0.032	>0.999	0.192
Visual retention	0.028	>0.999	0.316	>0.999	>0.999	0.388	0.023
PGI-MS Total Score	<0.001	>0.999	0.005	0.036	0.001	0.162	<0.001
Working Memory	0.017	0.145	>0.999	0.664	0.032	>0.999	0.192
T-score word by deviation score	0.028	>0.999	0.997	>0.999	0.052	>0.999	0.064
T score colour-word by deviation score	0.027	>0.999	0.118	>0.999	0.037	>0.999	0.143

In terms of pre and post values comparisons within the group, the study revealed that at post intervention only 5% participants had low level memory (score < 75) while the baseline details showed that 26.66% having the same. Similarly, 8.33% had below average levels (score 76 to 81) memory at post intervention as compared to 16.66% at baseline. The frequency of participants with excellent memory (score 80 to 100) increased from 5% to 46.66%, post intervention.

There was a significant effect of intervention on attention and concentration and Immediate Recall in group A (*p* = 0.003), B (*p* = 0.002), C (*p < 0*.*001)* and A (*p* = 0.005), B (*p*<0.001), C (*p* = 0.001) respectively. Similarly, the domains such as delayed recall and verbal retention of similar pairs were found to be significantly improved in group B (*p* = 0.041), C (*p* = 0.043) and A (*p* = 0.019), B (*p* = 0.041) respectively (Table 3). There was significant improvement in the verbal retention of dissimilar pairs in group A (*p* = 0.001), B (*p* = 0.039) and C (*p* = 0.001) (Table 3). A statistically significant effect of intervention was also observed in the visual retention domain in all groups except the controls. The improvement in the mean score was the greatest in group C (22.7%) followed by group A (19.06%), and group B (8.21%) (Table 3).

**Table 3:** Effect of intervention within the group.

Variables	Group A				Group B				Group C				Group D			
	Pre	Post	*p*-*value*	% change	Pre	Post	*p*-*value*	% change	Pre	Post	*p*-*value*	% change	*Pre*	*Post*	*p-value*	% change
Remote Memory	6.73 ± 0.79	6.93±0.26	0.189		6.73 ±0.59	7±0.0	0.104		6.8 ±0.77	6.93±0.26	0.334		6.73 ± 0.59	6.73±0.70	>0.999	
Recent Memory	4.87±0.35	5±0	0.164		4.73±0.70	4.93±0.26	0.189		4.93±0.26	5±0	0.334		4.73±0.59	4.8±0.56	0.334	
Mental balance	7.6±1.59	8.66±0.62	0.005	***13*.*95***	7.87±1.19	8.8±0.41	0.005	***11*.*82***	7.6±1.35	8.87±0.35	0.001	***16*.*71***	7.2±1.32	7.93±1.16	0.006	***10*.*14***
Attention concentration	6.93±2.15	9.93±3.77	0.003	***43*.*29***	9.73±4.37	11.67±4.22	0.002	***19*.*94***	8.93±2.96	14.6±1.96	<0.001	***67*.*52***	9.07±4.61	8.93±4.62	0.334	
Delayed recall	8.4±2.10	8.8±1.61	0.164		7.73±1.79	8.27±1.75	0.041	***6*.*99***	8.73±1.58	9.6±0.51	0.043	***9*.*97***	8.2±2.27	8.13±1.99	0.774	
Immediate recall	10.27±1.28	11.07±1.28	0.005	***7*.*79***	8.67±2.13	9.73±2.22	<0.001	***12*.*23***	9.8±2.14	11.4±1.24	0.001	***16*.*33***	10.13±1.13	10.13±1.36	>0.999	
Verbal Retention for similar pairs	4.6±0.63	4.93±0.26	0.019	***7*.*17***	4.6±0.74	4.87±0.35	0.041	***5*.*87***	4.73±0.59	4.93±0.26	0.082		4.530± 0.74	4.73±0.46	0.189	
Verbal Retention of dissimilar pairs	11.67±3.98	13.6±2.77	0.001	***16*.*54***	11.07±2.49	12.2±2.54	0.039	***10*.*21***	11.33±3.94	13.93±2.05	0.001	***22*.*95***	12.27±3.13	12.2±2.81	0.806	
Visual retention	9.13±3.40	10.87±2.39	0.006	***19*.*06***	10.6±1.72	11.47±1.51	0.004	***8*.*21***	10±3.16	12.27±0.88	0.004	**22.7**	9.67±2.61	10.13±2.50	0.068	
Recognition	9.8±0.41	10±0	0.082		9.2±1.26	9.6±0.83	0.028		9.93±0.26	10±0	0.3343		9.73±0.59	9.87±0.35	0.164	
Total score	80±7.91	89.8±7.51	<0.001	***12*.*25***	80.93±6.52	88.53±5.96	<0.001	***9*.*39***	82.8±7.34	97.53±2.74	<0.001	**17.79**	82.27±5.60	83.6±6.49	0.004	***1*.*62***
T-score word by deviation score	23.67±7.44	20.53±6.75	<0.001	***-13*.*2***	29.53±11.1	24.8±10.21	<0.001	***-16*.*02***	21.07±8.6	16±5.69	0.004	***-24*.*06***	27.53±11.65	24.53±11.43	<0.001	***-10*.*89***
T-score colour by deviation score	25.33±12.46	21.07±12.19	<0.001	***-16*.*82***	32.27±12	27.93±11.96	<0.001	***-13*.*45***	24.73±8.51	18.73±9.24	<0.001	***-24*.*26***	23.47±8.83	20.6±8.98	<0.001	***-12*.*23***
T-score colour- word by deviation score	37.6±7.62	34±7.3	<0.001	***-9*.*5***	38.47±7.27	35.13±7.26	<0.001	***-8*.*68***	33.67±6.61	27.87±6.17	<0.001	***-17*.*23***	36.73±6.35	33.8±6.46	<0.001	***-7*.*98***

As assessed by the PGI Memory scale, there was a significant effect of intervention on the mental balance, in group A (*p* = 0.005), B (*p* = 0.005), C (*p* = 0.001) and D (*p* = 0.006). However, the % change in the mean score of mental balance was found to be the greatest in group C (16.71%) (Table 3).

**Fig 2:**
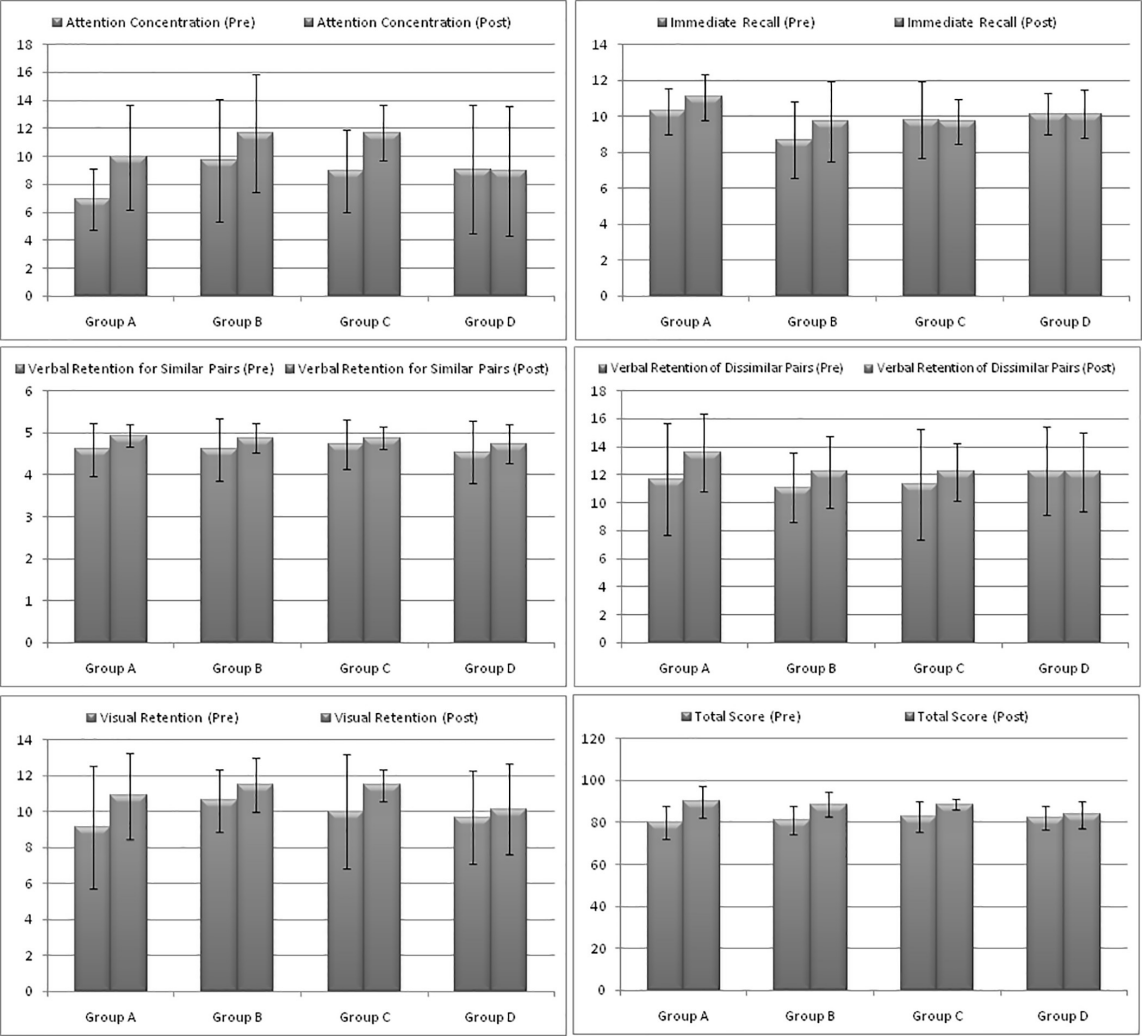
Average changes in cognitive functions from the baseline (Mean ±SD).

The pre-test-post-test change in the total PGI-MS scores indicated a statistically significant improvement in all groups including controls as compared to the baseline (Table 3 & Fig 2). The improvement in the mean PGI total score was 17.79% greater in group C, 12.25% in group A and 9.39% in group B when compared to the control group (1.62%) (Table 3).

There was a significant effect of intervention on working memory in group A (*p* = 0.005), B (*p*<0.001), and C (*p* = 0.001) and the improvement in the mean score was greater in group C as compared to group A and group B (Table 3).

The pre-test-post-test change in the visuo-spatial memory scores indicated a statistically significant improvement in group A (*p* = 0.006), B (*p* = 0.004) and C (*p* = 0.004). The details on within the group comparisons have been presented in Table 3.

#### Stroop Color Word Test

Among the study group, an overall significant difference was observed in the “T-score word by deviation” score (*p = 0*.*028*). But the difference was significant only between groups B and C (*p* = 0.052). Similarly, the overall T-score colour word by deviation score was found to be overall statistically significant (*p* = 0.027) and that existed between group B and C *(p* = 0.040) only (Table 2). However, within the group analysis revealed a statistically significant decrease in the T score word by deviation, as assessed by Stroop Color Word Test, in group A (*p*<0.001), groups B (*p*<0.001) C group (*p*<0.001*)* and group D (*p*<0.001*)*. While the highest decrease in mean was found to be in group C (-24.06%), and the least was seen in group D (-10.89%). Similarly, the T Score for colour and colour-word deviation was also found to be significantly reduced in all the four groups, having the highest decrease in group C and lowest in group D (Table 3). The details on the comparisons between the group and with-in the group have been presented in Tables 2 and 3, respectively. Average changes in cognitive functions of the study participants from the baseline (Mean ±SD) have been depicted in Fig 2.

### Compliance to the intervention

Participants were 100% compliant to the diet, physical exercise and CBCT. No adverse effect was reported during the trial.

## Discussion

The Multimodal Intervention in Subjective Cognitive Impairment (MISCI) trial is the first Pilot- RCT of its kind to study the effect of computer based cognitive training (CBCT), using RehaCom Software, as a single intervention or along with customized Mediterranean equivalent diet (MED) and or exercise (E) for six months on older adults with subjective cognitive impairment (OA-SCI). SCI, being the earliest stage in the paradigm of dementia, serves as the opportunity for the clinicians to intervene and prevent further progression.

In the present study, impairment was noted in various domains like attention and concentration, mental balance, retention of similar and dissimilar pairs in OA-SCI, at baseline. The MISCI trial showed that the multimodal therapy (CBCT+MED+E = CDE) was superior to unimodal intervention using CBCT. However, intervention with CBCT alone was also beneficial in mental balance, attention and concentration, immediate recall, verbal retention of similar and dissimilar pairs, visual retention and total PGI-MS score. The FINGER trial [13] has also noticed the superiority of multimodal intervention, but on participants with mild cognitive impairment (MCI).

Immediate recall, a component of short‑term memory, working memory and executive functions of an individual, is one of the earliest domains to be affected during the process of cognitive decline. The MISCI trial showed improvement in immediate recall in the individuals who intervened with CBCT and in the CDE groups which is in line with the observations by Yang et al [5]. However, Yang et al conducted their study on subjects with MCI. On delayed recall subscale of the PGI-MS, multimodal therapy was found to be superior to CBCT alone. Our findings are mirroring the observations by Cavalloet al [17] and Nousia et al [18], which showed the role of CBCT in the early stage of Alzheimer’s disease. The MISCI trial and its findings corroborate further that SCI is the precursor of MCI and other advanced stages of cognitive impairment with similar responses to non-pharmacological therapy.

Improvement was also noted in verbal retention for dissimilar pairs, in all intervention groups and the controls. However, the greatest improvement was observed in multimodal intervention groups, corroborating earlier reports [13].

The present study showed a significant improvement in mental balance and total PGI score, both in the intervention and control groups, but there was a greater improvement in the intervention group than in the control group. As witnessed by Yang et al [5] the present study also revealed improvement in both word and colour, as assessed by stroop test, in the intervention and control group. Improvement in the control group could be attributed to their adherence to brain stimulating activities like practicing Sudoku, mental math, learning new skills etc.

As reported previously [19, 20] the MISCI trial also revealed a significant improvement in working memory of the participants in all the three intervention groups.

Episodic memory, a subtype of long-term memory, helps to remember past events as well as details about the context (e.g. times, places, persons) [21]. Studies on the effect of cognitive therapy on episodic memory are often contradictory. While some studies [22, 23] confirmed beneficial effects of computer-based intervention programs on cognitive functions such as episodic memory or abstract reasoning, other studies [24, 25] revealed no effect of this intervention on cognitive functioning among older adults with MCI and/or dementia The finding of the present study is in correlation with the later as we could not establish any significant improvement in episodic memory of the study participants. The small sample size and the difference in the stage of intervention could be the reasons for this finding in our study.

Visuospatial abilities play a fundamental role in everyday activities but decline faster and earlier in Alzheimer’s disease (AD) [26]. However, neuropsychological research on AD hardly paid any attention to visuospatial memory. The present study showed a significant improvement in visuo-spatial memory of the study participants as compared to the control group, in agreement with the review by Kueider et al [27].

The MISCI trial not only showed the feasibility of computer-based cognitive training for Indian older adults but also their adherence to Mediterranean equivalent diet and exercise. It also revealed the superiority of multimodal intervention to improve the cognitive status of OA-SCI, as assessed by PGI-MS. The CBCT performed alone was also beneficial in improving cognitive status in OA-SCI. The MISCI trial would encourage the researchers to further work on simplified computer-based training protocol, especially smartphone based cognitive training which could be scalable to the masses. However, the availability of smart phones to all sectors of older people and the digital divide are some concerns.

### Strength and limitations

The MISCI trial is the first of its kind RCT where older adults with subjective cognitive impairment were intervened with a multimodal approach using computer-based cognitive therapy, MED, and customized exercise regimes. Small sample size and non-generalizability being a unicentric study were some of the limitations.

## Conclusion

The MISCI trial showed the positive effects of multimodal non-pharmacological intervention with computer based cognitive training, Mediterranean equivalent diet and exercise on various cognitive domains in older adults with subjective cognitive impairment. The present study gives an indication that an early intervention at SCI stage would prevent or halt the progress of cognitive impairment among older adults. Multicentric smartphone based cognitive training can be tried to reach the masses with SCI.

## Supporting information

S1 Checklist TREND statement checklist. (DOCX)

S1 Protocol (DOCX)

S1 Data (XLSX)
