# Supplementary material for: Effect of Multimodal Intervention (computer based cognitive training, diet and exercise) in comparison to health awareness among older adults with Subjective Cognitive Impairment (MISCI-Trial)—A Pilot Randomized Control Trial
Source: PLoS One. 2022 Nov 3;17(11):e0276986. doi: 10.1371/journal.pone.0276986 (PMC9632783; doi:10.1371/journal.pone.0276986)
Supplement: S1 Protocol — (DOCX) [file pone.0276986.s002.docx]

**Multimodal Intervention using a combination of computer-based cognitive training, dietary counselling, and exercise therapy in Indian elderly having Subjective Cognitive Impairment: A Randomized Controlled Trial**

**Background**

Subjective cognitive impairment refers to self-reported deterioration in cognitive performance, which is not identified objectively, by means of neuropsychological testing. Recent epidemiological evidence suggests that elderly with subjective cognitive decline, are at a heightened risk of developing dementia. Additionally, it has been reported that this population group has a relatively greater prevalence of biomarkers for amyloidosis, as well as existence of neuro-degeneration. Nonetheless, Alzheimer's disease is not the cardinal factor contributing to subjective cognitive impairment, and multiple other conditions are typically associated with subjective memory complaints, particularly, some psychiatric disorders and normal process of aging.

**Aim**

To study the effect of multimodal intervention, including cognitive training using RehaCom software, dietary counselling, and physiotherapy on improvement of cognitive function and cognitive performance in elderly, diagnosed with subjective cognitive impairment in community settings and out-patient clinics.

**Methods**

**Study Design**

A randomised controlled trial would be carried out by recruiting elderly visiting the out-patient-clinic. Subjects will be randomized 1:1:1:1 to receive either computer based cognitive therapy (CBCT) based on a Germen software Rheacom, CBCT+ diet which will be created after modification of Mediterranean diet or CBCT+Diet+ Exercise regime (which will be created by experts in the field of Physiotherapy) and the control group. The intervention group will receive 6 months of intervention to have 40-44 sessions, of 40 minutes duration and the control group brain stimulating activities such as sudoku, mental maths, and learning music and new skills.

**Study Population**

Inclusion criteria are as follows: all consenting elderly subjects visiting the out-patient-clinic of AIIMS having SCI, aged >60 years. Both elderly men and women will provide written or verbal consent to participate in the trials.

**Intervention**

The group imparted with computer-based customized training using RehaCom software will be assessed using Neuropsychological Test Battery (NTB) including, PGI- Memory Scale, Wisconsin Card Sorting Test, Stroop Color and Word Test. Stroop Color and Word Test will assess the relative speed of reading the names of colors and identifying the colors correctly that are printed in another color. It comprises of an interference component because it requires the participant to exhibit a reading response. The advantage of using this test is that it measures the ease with which a person can shift his perceptual set to adapt to changing demands, and to inhibit the usual response from interfering with the unusual one. This test is a measure of two essential characteristics: cognitive flexibility and response inhibition. Moreover, the PGI memory scale will be used to measure verbal and nonverbal memory. The ten sub-tests measure different memory domains such as, remote memory, recent memory, mental balance, attention and concentration, delayed recall, immediate recall, retention for similar pairs, retention for dissimilar pairs, visual retention, and recognition. Furthermore, cognitive training will include assessment of abstract reasoning and mental flexibility using the Wisconsin Card Sorting Test. The subjects can classify cards according to the color, shape of its symbols, or the number of the shapes shown on each card. With a change in the classification rule every 10 cards, it is expected that once the participant has figured out the rule, he/she will start making mistakes with an alteration in rules. This task fundamentally measures how well people can adapt to the changing rules.

Comprehensive geriatric assessment will be carried out using the Charlson Comorbidity Index, the Barthel ADL Index, IADL Scale, the Kuppuswamy scale for socio-economic status, GDS-15 for assessment of depressive symptoms, Mini-Cog for cognitive functioning, and ICIQ for determining the presence of urinary continence in subjects.

The exercise therapy treatment group will undergo training using Short Physical Performance Battery which comprises of balance, gait speed, and chair stand tests. Each training session will last for a minimum of sixty minutes. Balance tests such as, side-by-side stand, semi-tandem stand, tandem stand will be carried out. Moreover, gait speed testing will be carried out to assess abnormalities or deviations in normal gait pattern. Chair stand tests will also be conducted to assess leg muscle strength. Furthermore, subjects will undergo resistance training exercises involving shoulder front press, shoulder side raises, biceps curls, triceps extension, hams curls, dynamic quads, supine SLR, wall slides, and abdominal curls for three days in a week and this treatment will continue for a period of six months. During the first month of exercise training, subjects will be asked to perform (8-15) × 2 repetitions with a maximum load of 40-50% RM and the repetitions will increase to 20 × (1-3) with maximum load of 70-80% RM.

The dietary intervention will be customized from FINGER trial for Indian population in a small sample.

Then the customized diet plan focusing on increasing consumption of foods rich in omega-3 fatty acids such as flaxseeds, flaxseed oil, walnuts, mustard oil, soybean oil, roasted soybeans etc.

At baseline, patients will be assessed using the Mini Nutritional Assessment (MNA) for determining their nutritional risk level. A dietary profile questionnaire will also be administered to elderly which will gather information on their meal and snacking patterns, oral health problems, intake of omega-3 dense food items, and quantity and quality of cooking oil used in their household. Food frequency questionnaire will be used to collect information on the frequency of foods regularly consumed by the elderly subjects in the past six months.

**Outcomes**

The primary outcome will be measured after 6 months by evaluating change in cognitive performance by using PGI Memory Scale total score. Improvements in cognitive domains including executive functioning, processing speed, attention, and memory will be recorded. Secondary outcomes including change in functionality and quality of life will be measured using Barthel's ADL Index, Lawton's Scale, and SF-36 respectively. By utilization of the visual paired associates test, immediate and delayed recall, logical memory immediate and delayed recall, word list learning and delayed recall will be reassessed for noticing changes in the memory domain. Adverse events such as presence of psychological stress, musculoskeletal pain, or injury will be recorded. An external safety committee will regularly assess safety-related issues throughout the duration of the study.

**Sample Size**

Since it is a pilot study, 15 participants will be recruited in each group as per feasibility. Considering 10% dropout due to loss to follow up, 17 participants will be recruited in to each group giving a total of 68 participants.

**Analysis Plan**

Data will be entered and analyzed using STATA software. Neuropsychological Test Battery (NTB) total score and the z-scores for executive functioning, processing speed, and memory will be calculated. For computing the NTB total score, at least 8/14 NTB components will be needed; at least 3/5 test scores for the executive functioning domain, 3/6 test scores for the memory domain, and 2/3 test scores for evaluating processing speed. Mixed-model repeated-measures analyses having a maximum likelihood estimation will be used to analyze changes in cognitive performance as a function of randomization group (dichotomous variable coded as 0 for control and 1 for intervention), time (continuous variable coded as 0 for baseline, 6-month visit, characteristic, and group × time × characteristic interaction. The characteristics will be either dichotomous (sex, presence of nutritional risk, and deterioration in quality of life) which will be dichotomized depending on the median value) or continuous variables (age, education level, scores on the following scales: MMSE, Mini-Cog, BADL, IADL, GDS-15, SF-36, and MNA). Log-transformation will be appropriately applied to the skewed continuous variables.
